# mHealth-supported tuberculosis contact investigation by community health workers in high-burden countries: a scoping review

**DOI:** 10.1080/16549716.2026.2684132

**Published:** 2026-06-09

**Authors:** M. Dody Izhar, Abdul Wahab, Wahyudi Istiono, Yodi Mahendradhata

**Affiliations:** a Department of Epidemiology, Faculty of Medicine and Health Sciences, Universitas Jambi, Jambi, Indonesia; b Doctoral Program in Medical and Health Sciences, Faculty of Medicine, Public Health and Nursing, Universitas Gadjah Mada, Yogyakarta, Indonesia; c Department of Biostatistics, Epidemiology, and Population Health, Faculty of Medicine, Public Health and Nursing, Universitas Gadjah Mada, Yogyakarta, Indonesia; d Department of Family and Community Medicine, Faculty of Medicine, Public Health and Nursing, Universitas Gadjah Mada, Yogyakarta, Indonesia; e Center for Health Policy and Management, Faculty of Medicine, Public Health, and Nursing, Universitas Gadjah Mada, Yogyakarta, Indonesia

**Keywords:** Implementation science, health system, digital health, technology acceptance, high-burden settings

## Abstract

Mobile health (mHealth) implementation strategies in tuberculosis (TB) contact investigations are intended to increase community health workers’ (CHWs) engagement in TB contact investigations and case detection. This scoping review aimed to map and synthesize existing evidence on the design and development of acceptable and feasible mHealth implementation strategies for CHW-led TB contact investigations. We conducted a scoping review following the Arksey and O’Malley framework and PRISMA-ScR guidelines. We searched PubMed, Scopus, Cochrane Library, Web of Science, and Google Scholar for English-language studies published through October 2024 on mHealth interventions for TB contact investigation by CHWs. Eligible studies included empirical studies conducted in high-burden countries (HBCs) for TB, HIV-associated TB, and MDR/RR-TB. Studies were screened, and data were extracted using Rayyan AI-assisted software. We mapped implementation strategies, barriers, facilitators, acceptability, and feasibility of mHealth interventions. Quantitative findings were described, and qualitative data were synthesized thematically. Nine studies were included. Most interventions used hybrid mHealth technologies for communication, case registration, health records, and decision support. Implementation strategies specified actors, actions, targets, temporality, dose, and justifications. Barriers were related to CHWs, digital systems, service implementation, and TB-affected persons. Facilitators included stakeholder support, CHW training, mHealth design, community engagement, and patient-centered approaches. Overall, CHWs perceived mHealth interventions as acceptable and feasible. Successful implementation in HBCs requires strengthened CHW capacity, reliable technology, optimizing interaction between CHWs and digital tools, and continuous monitoring and evaluation throughout implementation.

## Paper Context

- **Main findings:** Health interventions for tuberculosis contact investigation by community health workers in three global lists of high-burden countries for tuberculosis, human immunodeficiency virus-associated tuberculosis, and multidrug-resistant/rifampicin-resistant tuberculosis are generally acceptable and feasible, but their effectiveness depends heavily on a clear implementation strategy and the ability to address multilevel barriers at the individual, technology, and service system levels.
- **Added knowledge:** Given the ongoing development of digital health interventions for TB contact investigation by community health workers, this review provides a novel contribution by systematically mapping seven dimensions of implementation strategies, classifying barriers and facilitators at multiple levels, and highlighting the importance of synergies between human actors and digital systems in determining implementation success.
- **Global health impact for policy and action:** These findings confirm that strategic integration of mHealth into national tuberculosis policies and programs – with sustained funding, community health worker capacity building, digital system interoperability, and implementation-based monitoring – is crucial for closing the contact investigation gap and accelerating the achievement of the global tuberculosis elimination target.

## Background

Globally, the estimated incidence rate of TB was 134 per 100,000 (95% UI: 125–145) in 2023. The net reduction in the TB incidence rate from 2015 to 2023 was 8.3%, well below the World Health Organization (WHO) End TB Strategy’s milestone of a 50% reduction by 2025 [1]. The WHO has called for a focus on global action in 49 countries included in three global lists of high-burden countries (HBCs) for TB, human immunodeficiency virus (HIV)-associated TB, and multidrug-resistant/rifampicin-resistant TB (MDR/RR-TB), which together account for nearly 90% of the estimated global incidence [1].

Active case finding (ACF) among TB contacts in healthcare and community settings is crucial [2–5]. It helps close the gap of approximately 3.1 million undiagnosed and underreported cases worldwide [1,6–8]. The WHO recommends household contact investigation as a routine practice in low- and middle-income countries (LMICs) [8]. The ACF of TB in HBCs is very effective with the adoption of a contact investigation strategy [9,10]. Contact investigations systematically identify active and latent TB, thereby identifying eligible candidates for and facilitating the initiation of preventive therapy [8,11].

Community-based contact investigations, specifically those led by or involving CHWs, are critical elements in achieving the end of the TB epidemic [12,13]. The involvement of CHWs in community-based contact investigations serves to address gaps in health service access, particularly for marginalized communities facing the challenges of chronic infectious diseases like TB [14]. This engagement enhances surveillance and trust [15], acting as a vital liaison between society and services to achieve Universal Health Coverage (UHC) under Sustainable Development Goal (SDG) 3.3 [6,16,17]. CHWs support contact investigation and ACF activities through coordination with health service providers [12,18,19].

However, CHWs continue to face several barriers in conducting TB contact investigations, including difficulties visiting patients and contacts [20], stigma [21], limited transportation [12,19], fear of being exposed to TB [22,23], limited data because recording and reporting are performed manually [24,25], inadequate incentives [26,27], and limited coordination with health service providers [25,28]. These problems directly impact the lack of information or case index data, making it difficult to track and find TB-affected persons, minimal educational activities, or even no activities at all, resulting in low achievement of TB case-finding targets in the community [25,28].

To address these barriers, mHealth has emerged as a new approach with significant potential to help overcome efforts in TB prevention, detection, treatment, and management [29]. Digital technology can strengthen healthcare systems [30] while still adapting to local and national guidelines [31]. Based on the Classification of Digital Interventions, Services, and Applications in Health (CDISAH), digital functionality meets needs or overcomes issues in the health service system, and types of applications and services are used [32].

Given the potential of digital technology interventions for TB contact investigation involving CHWs, and the limited availability of mHealth interventions in HBCs [29,31], as well as the persistence of real-world barriers to interventions that contribute to the lack of acceptability and feasibility of mHealth implementation [33,34]. Therefore, it is necessary to identify and map previously implemented mHealth interventions through a scoping review across HBCs. This scoping review aims to map mHealth interventions, implementation strategies, barriers, and facilitators to inform the design of acceptable and feasible mHealth programs led by CHWs in HBCs.

## Methods

### Protocol and registration

This scoping review protocol was registered on 7 October 2024, under the Open Science Framework (OSF) (https://doi.org/10.17605/OSF.IO/M3A8V).

### Study design

This scoping review followed the framework established by Arksey and O’Malley [35], enhanced by Levac et al. [36], and the Joanna Briggs Institute’s systematic scoping review guide [37,38]. The process comprised six stages: (1) identify the research questions; (2) identify relevant studies; (3) study selection; (4) chart the data; (5) collate, summarize, and report the results; and (6) consultation. The review was conducted according to a predefined protocol and is reported in adherence with the Preferred Reporting Items for Systematic Reviews and Meta-Analyses extension for Scoping Reviews (PRISMA-ScR) (see Supplementary Material 1) [39].

### Research questions

1. How was the mHealth implementation strategy used in TB contact investigations by CHWs?
2. What barriers and facilitators affect the implementation of mHealth in TB contact investigations by CHWs?
3. What are the acceptability and feasibility of mHealth implementation strategies in TB contact investigations by CHWs?

### Data sources and search strategy

Based on the research question and the eligibility criteria for the studies to be reviewed, we used the Population, Intervention, Comparison, and Outcome (PICO) framework. The population for this scoping review was CHWs or other relevant terms used in the literature who conducted TB contact investigations. The CHWs population was based on the definition of implementation research (IR), that is, ‘who’ refers to those who manage and implement the intervention, and allows for a combination with public servants and the general public [40]. However, a comparison group was not required, given that the purpose of this review was to map the implementation landscape (see Supplementary Material 2).

Furthermore, several relevant studies were selected. The literature search strategy used the Peer Review of Electronic Search Strategies (PRESS) [41], applied by one author (MDI) and one academic librarian from Universitas Gadjah Mada, to the electronic databases PubMed, Scopus, the Cochrane Library, Web of Science, and additional records identified through Google Scholar searches.

The pilot search was conducted in October 2024. The search was limited to papers published in English without year restrictions. A standardized set of search terms consisting of ‘community health workers’, (‘contact investigation’ OR ‘contact tracing’), ‘tuberculosis’, ‘mobile health’, and (feasibility OR acceptability OR effective*) was used for both the electronic databases and Google Scholar. The search was conducted independently by two authors (MDI and WI), resulting in 576 records from electronic databases and 263 additional records from Google Scholar to complement the search (see Supplementary Material 3).

### Study selection

Our initial search results from electronic databases and supplementary records included 839 papers, which were then entered into the new.rayyan.ai software [42] for a three-stage selection process (title, abstract, and full text) conducted by two authors (MDI and AW). A senior reviewer (YM) provided input during the screening, data extraction, and analysis stages and provided critical comments on the manuscripts. Of the 839 papers, 751 were included for screening after 88 duplicates were removed.

For inclusion, empirical studies published up to the time of the search, all types of study designs, English-language articles published in journals and gray literature, focused on the acceptability and feasibility of mHealth implementation for CHW-led TB contact investigation in HBCs, and met the predefined population, concept, and context criteria (see Supplementary Material 4). Papers published in the form of discussions, opinions, theses, letters to the editor, conference abstracts, and comments were excluded.

**Figure 1.**
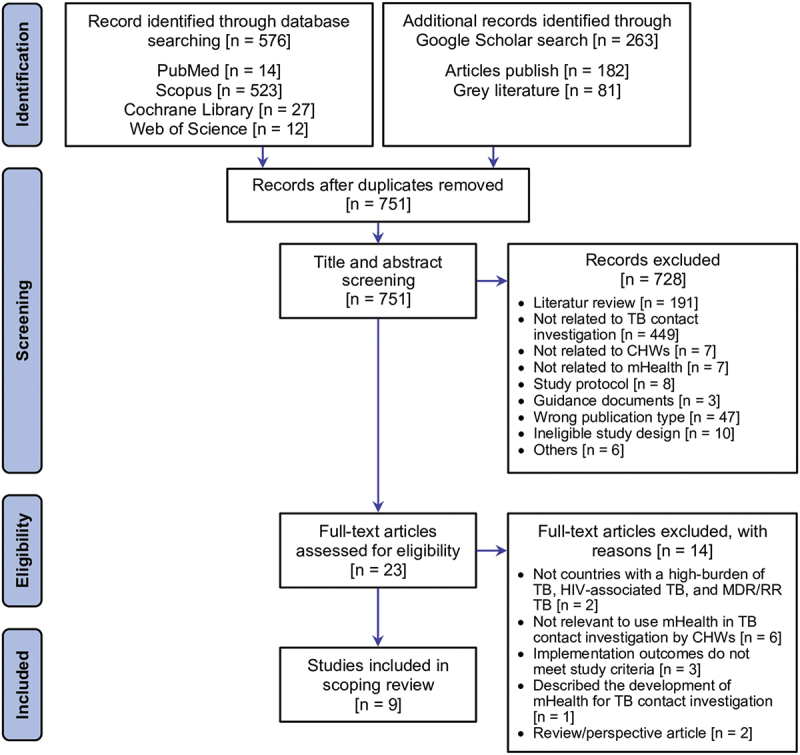
PRISMA flow diagram.

From the screening results, 728 papers were excluded based on title and abstract review because they were not relevant to the research question, did not meet the inclusion criteria, or were included in the exclusion criteria. The final selection stage, from the full-text review (*n* = 23), was conducted to evaluate eligibility, and 14 papers were excluded. Finally, the selected papers (*n* = 9) were included in the scoping review and exported to Mendeley V1.19.8. (List of databases in RIS format). The entire selection process and the reasons for exclusions are documented in Figure 1.

### Data charting and synthesis

All studies included in the scoping review were subsequently subjected to data extraction. Before the data extraction process, the variables for extraction were agreed upon by all the authors (see Supplementary Material 5).

Extracted data were synthesized and reported according to the IR framework for mHealth use in CHW-led TB contact investigation, including (1) mHealth implementation strategies based on dimensions such as the actor, the action, action target, temporality, dose, and justification; (2) barriers and facilitators of mHealth implementation; and (3) implementation outcomes: acceptability and feasibility. The results of the quantitative analysis are presented descriptively through tables, frequency distribution tables, and statistical summaries. In qualitative analysis, we synthesize findings thematically and transform them into appropriate text units.

### Consultation

Based on sufficient study evidence from the scoping review, we further consulted with TB program managers at the district and provincial levels in Jambi Province, Indonesia, as well as the Doctoral Program in Medical and Health Sciences, Faculty of Medicine, Public Health, and Nursing, Universitas Gadjah Mada, to obtain additional advice and information to design and develop an evidence-based and contextual mHealth implementation strategy in TB contact investigation involving CHWs, which can serve as a pilot for the future.

### Results

In total, nine papers were included in this scoping review. Articles were published between 2014 and 2023, and most (*n* = 6) were conducted in sub-Saharan Africa (e.g. Botswana, Kenya, and Uganda) and parts of Asia (*n* = 3) (e.g. Cambodia, Pakistan, and Indonesia) (Table 1).

**Table 1.** Characteristics of studies included.

Title	Authors [Years]	Country [Setting]	Aim	Study design/type of study	Type of CHWs	mHealth technology
Home-based tuberculosis contact investigation in Uganda: a household randomised trial.	J. Lucian Davis et al. [34] [2019]	Uganda [Urban]	To determine whether home-based strategies and SMS-facilitated approaches can increase the proportion of contacts who complete TB evaluation and receive new TB diagnosis and treatment.	Household-randomised trials study/ quantitative	CHWs	Mobile app + SMS
Experiences and intentions of Ugandan household tuberculosis contacts receiving test results via text message: an exploratory study.	Joseph M. Ggita et al. [49] [2020]	Uganda [Urban]	To explore how household contacts experience test results delivered via SMS, and how these experiences influence follow-up intentions.	Exploratory study/ qualitative	LHWs	Mobile app + SMS
Community-based active tuberculosis case finding in poor urban settlements of Phnom Penh, Cambodia: a feasible and effective strategy.	Natalie Lorent et al. [43] [2014]	Cambodia [Urban poor]	To assess the feasibility of community-based ACF for TB among the urban poor in Cambodia and determine its impact on case detection, treatment uptake, and outcome.	Survey/ quantitative	CHVs and HCWs	Mobile phone + SMS
Feasibility, acceptability, and adoption of digital fingerprinting during contact investigation for tuberculosis in Kampala, Uganda: a parallel-convergent mixed-methods analysis.	Elizabeth B White et al. [33] [2018]	Uganda [Urban]	To understand the feasibility, acceptability, and adoption of digital fingerprinting for patient identification in a study of household tuberculosis contact investigation in Kampala, Uganda.	Mixed-methods study/ quantitative and qualitative	CHWs	Biometrics
Factors affecting the transition from paper to digital data collection for mobile tuberculosis active case finding in low internet access settings in Pakistan.	Christina Mergenthaler et al. [44] [2022]	Pakistan [Remote/ low internet access]	To compare the quality of data entered in the respective (matching) CAPI and PAPI patient-based records, and evaluate users’ perceptions on feasibility and sustainability of the hybrid PAPI/CAPI approach and full CAPI approach in low internet chest camp settings.	Mixed-methods study/ quantitative and qualitative	CHWs, clinicians, supervisor, and medical doctor	Mobile phone/tablet app
Evaluation of a mobile health approach to tuberculosis contact tracing in Botswana.	Yoonhee P. HA et al. [45] [2016]	Botswana [Urban and suburban]	To develop and evaluate a mHealth approach that addresses many of the limitations of the paper form-based approach used in Botswana.	Design and development research/ quantitative	Caretakers and HCWs	Mobile phone/tablet app
Using a mobile application to improve pediatric presumptive TB identification in Western Kenya.	Daria Szkwarko et al. [46] [2021]	Kenya [Rural hospital]	To incorporate the CHV-led presumptive pediatric TB mobile android application (PPTBMAPP) at a rural hospital in Western Kenya to assess its feasibility, appropriateness, and effectiveness in increasing pediatric presumptive TB and TB disease detection.	Mixed-methods study/ quantitative and qualitative	CHVs	Mobile app
Development of the SIKRIBO mobile health application for active tuberculosis case detection in Semarang, Indonesia.	Sri Ratna Rahayu et al. [47] [2022]	Indonesia [Urban and suburban]	To document the development and usability testing of SIKRIBO, a tuberculosis screening application.	Design science research/ quantitative	Health cadres	Mobile app + website
mHealth to improve implementation of TB contact investigation: a case study from Uganda.	Amanda J. Gupta et al. [48] [2023]	Uganda [Urban]	To compare the implementation and effectiveness of standard, clinic-based TB contact investigations with the locally adapted interventions, home-based contact investigations, paired with the mHealth-facilitated implementation strategy.	Case study/ quantitative and qualitative	CHWs	N/A

TB: tuberculosis; SMS: short message service; CHWs: community health workers; app: application; LHWs: lay health workers; ACF: active case finding; CHVs: community health volunteers; HCWs: healthcare workers; CAPI: computer-aided personal interviewing; PAPI: paper and pencil interviewing; PPTBMAPP: presumptive pediatric TB mobile android application; N/A: not applicable.

### Study setting and design

Most studies were conducted in urban settings (*n* = 5) [33,34,43,48,49], with limited evidence from urban and suburban settings (*n* = 2) [45,47], rural areas (*n* = 1) [46], and remote-low internet access (*n* = 1) [44]. The most frequently used method is mixed-methods studies (*n* = 3) [33,44,46], and two studies use the design and development research approach (*n* = 1) [45] and a design science research approach (*n* = 1) [47], while the remaining studies used a survey approach (*n* = 1) [43], exploratory study (*n* = 1) [49], randomized household trial study (*n* = 1) [34], and case study (*n* = 1) [48] (see Table 1).

### Types of mHealth technology

Six studies (75%) used hybrid mHealth technologies that combined multiple digital components within their operational frameworks (e.g. mobile app + SMS, mobile phone + SMS, mobile app + web, and mobile phone/tablet app).

Based on the CDISAH [32], we identified four classifications of Digital Health Interventions (DHIs) as digital functionalities designed to address healthcare system challenges: 1) targeted communication to persons via mobile apps + SMS [34,49] and mobile phones + SMS [43], 2) identification and registration of persons using biometrics [33] and mobile phone/tablet apps [44], 3) person-centered health records via mobile phone/tablet apps [45], 4) healthcare provider decision support using mobile apps [46] and mobile apps + websites [47] (Table 1).

### Implementation strategy

In this scoping review, we explore seven dimensions of one of the prerequisite components for measuring implementation strategies [50], namely specifications, including actors, actions, action targets, temporality, dose, implementation outcomes, and justification in answering the first research question. The prerequisite components for measuring implementation strategy on the basis of CDISAH [32] (Table 2).

**Table 2.** mHealth implementation strategies and the implementation outcomes from the included studies.

	Implementation Strategy						Implementation Outcome	
Authors [years]	The actors	The actions	Action target	Temporality	Dose	Justification	Acceptability	Feasibility
***Targeted communication to persons [1.1]***								
J. Lucian Davis et al. [34] [2019]	CHWs (*n* = 14, trained, and reimbursed).	Mobile application intervention (CommCare Messaging) with SMS (one-way) compared to the standard-of-care approach. CommCare Messaging was used to deliver sputum test results, referrals for clinic follow-up, reminders, information, education, treatment evaluation for household contacts, and reports to health facilities and the NTP.	TB household contacts aged ≥ 15 years: Intervention arm (*n* = 471), and the standard-of-care (*n* = 448).	• July 2016 to July 2017. • The time required from sending an SMS message to household contacts until the TB evaluation is complete.	The intervention arm: Household contacts had a marginal probability of completed TB evaluation by day 14 of 14% (95% CI 8–20). The standard-of-care arm: Household contacts had a marginal probability of completing the TB evaluation on day 14 of 15% (95% CI 9–21).	CommCare messaging intervention is acceptable and feasible in TB contact investigation by CHWs, by ensuring phone ownership and confirmation of delivery and reading of messages among household contacts.	CommCare messaging was welcomed, liked, agreed upon, and accepted by CHWs. However, incomplete delivery of the intervention, low confirmation of mobile phone ownership, reading and receipt of SMS contributed to low completion and yield of TB contact investigations.	CommCare Messaging was easy to use for notification of sputum test results and follow-up instructions to TB household contacts, but was not feasible or significant in improving completion and yield of TB contact investigations, due to the complexity of implementation, lack of confirmation on mobile phone ownership, reading, and receipt of SMS (<20% achieved its full effect).
Joseph M. Ggita et al. [49] [2020]	LHWs.	Explored the feelings, beliefs, decisions, and behaviors of TB household contacts who received sputum test results sent via SMS.	TB household contacts who received negative TB results (*n* = 9) and positive TB results (*n* = 1).	October 2017 to November 2017.	Interviews were conducted for 30 to 45 minutes with ten household contacts 10 to 16 weeks after receiving their test results via SMS.	Notification of sputum test results via SMS should function as an instrument to complement ongoing interactions, and LHWs should continue to deliver results directly to household contacts to influence further care.	The SMS from LHWs about results and follow-up instructions received by household contacts was greeted with both relief and anxiety, and they stated that notification of sputum test results via SMS should complement direct interactions with LHWs, not replace them.	Not Stated.
Natalie Lorent et al. [43] [2014]	CHVs (*n* = 372, trained, intervention explanation, and incentive). HCWs (*n* = 37).	Mobile phone and SMS interventions to inform individuals with TB symptoms about positive TB diagnostic test results from door-to-door screening, referrals, treatment evaluation, and reporting to healthcare facilities and the NTP.	Individuals, aged ≥ 15 years (*n* = 315,874).	February 9, 2012 to March 31, 2013. The time from sputum collection until diagnostic test results are delivered via SMS.	Notification of positive smear microscopy/Xpert results was done promptly (within 24 hours). The median time from sputum collection to notification of the first positive result via mobile phone/SMS was 3 days, while the median time for written results was 5 days, and ≥94% initiating TB treatment and 81% completing it.	Notification of TB diagnostic test results via SMS improved access to timely TB diagnostic services, referral, follow-up, and completion or yield of TB screening.	Not stated.	The use of mobile phone/SMS in TB ACF involving CHWs to notify patients of positive TB test results was implementable and able to shorten the median time from sputum collection to notification of diagnostic results, reduce diagnostic delays, and increase initiation and completion of TB treatment.
***Identification and registration of persons [2.1]***								
Elizabeth B White et al. [33] [2019]	CHWs (*n* = 15, aged from 24 to 54 years, 80% secondary education, trained, and intervention training).	Performed offline multispectral fingerprint scanning (biometrics) on index TB patients and household contacts. Biometrics were used for record-keeping, avoiding duplicate registrations, ensuring data integrity, follow-up visits, and feedback. CHWs received training, courses, and role-plays on using biometrics and common problems.	TB household contacts aged ≥ 5 years (*n* = 919).	• July 2016 to July 2017. • The identification and registration of household contacts per contact was performed simultaneously with biometric tracking.	The median frequency of successful fingerprinting of all contacts in the household by CHWs was 71% (*p* < .001). However, over time (up to quarter 4), the proportion of households where all contacts were successfully fingerprinted decreased (*ρ*=.30, *p* < .001).	Biometrics were feasible and acceptable for the identification of household contact individuals in resource-constrained settings, with an emphasis on periodic process evaluation during implementation to assess effectiveness and guide delivery improvements.	Biometrics were accepted by CHWs for contact identification, like, appeal, useful, belief, optimism, work motivation, and enhancing their social and professional skills during the pilot study, but malfunctions with hardware and software reduced interest in using the technology and resulted in low fingerprint scanning rates.	Biometric was deemed feasible by CHWs with a 71% implementation success rate, easy to use, relevant to work, and smooth in preventing duplicate registrations, referrals, monitoring, and follow-up in TB contact investigations. However, over time, the proportion of households that were successfully fingerprinted tended to decline due to the malfunctioning of the technology.
Christina Mergenthaler et al. [44] [2022]	Clinician (*n* = 1), district field supervisor (*n* = 1), medical doctor (*n* = 1), CHWs (*n* = 1, implementation training).	The project intervened and evaluated the use of CAPI offline to replace PAPI for identification and registration during individual screening for presumptive TB in chest X-ray camps. CAPI supports TB ACF in remote areas with low internet access for data integrity, data sharing, and feedback to the NTP.	Individuals were screened and registered using PAPI (*n* = 6,876) and CAPI (*n* = 6,922).	• August 25, 2020, to March 27, 2021. • Completeness, accuracy, and consistency of data recording from symptomatic participants to the start of treatment for those diagnosed with TB.	Participants in chest X-ray camps through offline CAPI had a ratio of 1.01 (95% CI: 0.90–1.12) compared to the hybrid CAPI/PAPI approach (*n* = 1,505) with a ratio of 2.35 (95% CI: 1.30–3.41), with higher data completeness ratios for participant enrollment and people with presumptive TB, while accuracy and consistency had similar and varying proportions. User acceptance of CAPI was based on ease of use, efficiency, and safety principles.	The CAPI tool was an acceptable and feasible tool in TB ACF in remote areas and low internet access, considering interventions to include record-linking technology, clear protocols, supervision, and data accompaniment.	The intervention of CAPI was acceptable for data recording and completeness in TB ACF, and as a tool that was easy to use, convenient, safe, and effectively managed its uses, and useful, such as recording, collection, delivery of examination results, treatment initiation, monitoring, and evaluation.	The intervention of CAPI was generally feasible to be used to improve the recording and completeness of individual data in TB ACF, although data accuracy and consistency had similar or varying proportions with PAPI. CAPI was used to record, collect, send test results, initiate treatment, monitor, evaluate, and share data with NTP.
***Person-centered health records [2.2]***								
Yoonhee P. HA et al. [45] [2016]	Caretakers’ adult and pediatric TB cases, and male HCWs (*n* = 2),	Designed and developed a mobile/tablet app using ODK and HTC Flyer^TM^ to replace paper-based contact tracing. Evaluated the completion time for tracking, the generation and access of summary data, data quality, longitudinal tracking of individual health status and services, and user satisfaction with the CSUQ.	TB household contacts were screened using mHealth (*n* = 307) and paper-based (*n* = 170) methods.	mHealth-based: September 18, 2012 to March 17, 2013. Paper-based: March 18, 2012, to September 17, 2012. Time required to complete TB contact tracing.	Median tracking completion times for mobile/tablet app-based vs paper-based: adult TB cases (2.8 minutes vs 5.0 minutes), pediatric TB cases (3.2 minutes vs 5.0 minutes). Mobile/tablet apps were favorable as a contact tracing instrument (2.1/7.0).	Mobile/tablet apps were acceptable and feasible to replace paper-based TB contact tracing in improving trace completion time and data quality for TB control in resource-limited settings.	The intervention of the mobile/tablet app was acceptable for TB contact tracing, and it stated that the app was attractive, liked, welcomed, useful, and appropriate.	The intervention of household contact tracing using a mobile/tablet app was shown to be feasible and easy to use, reducing contact tracing completion time, and improving data quality compared to a paper-based form approach (*p* < .001) in resource-limited settings.
***Healthcare provider decision support [2.3]***								
Daria Szkwarko et al. [46] [2021]	CHVs (*n* = 7, trained).	The PPTBMAPP intervention for presumptive TB screening. After CHVs upload data, the app automatically notifies children for referral and follow-up TB testing if they meet presumptive TB. The app is easy to use, reduces the workload of HCWs, provides notification, communication, and screening feedback for CHVs, and reminds HCWs of the CHVs’ role.	Children aged ≤ 15 years (*n* = 1,787).	Pre-intervention: August 2018 to January 2019. Post-intervention: August 2019 to January 2020. The app automatically notifies that the child meets the criteria for presumptive TB.	The PPTBMAPP intervention significantly increased the proportion of presumptive pediatric TB between the pre- and post-periods (*p* = 0.0005) and referrals of presumptive pediatric TB (*p* = 0.00013), but was not statistically significant in the proportion of pediatric TB disease notifications (*p* = 0.5).	CHV-led PPTBMAPP is feasible to provide decision support to CHVs on TB presumptive detection, ease of use, notification, facilitating referral and follow-up of pediatric TB cases to clinics, communication, feedback for CHVs, CHV roles, and reducing HCWs’ workload.	Not stated.	The PPTBMAPP intervention led by CHVs was feasible for increased presumptive TB screening in children aged ≤ 15 years, ease of use, communication, linkage to TB clinics, and reduced the workloads of HCWs, such as registration, follow-up, reminders, referrals for pediatric TB cases, and reporting.
Sri Ratna Rahayu et al. [47] [2022]	Health cadres. (*n* = 20, aged from 17 to 56 years, 70% graduates, trained, intervention training).	Conducted development, interventions, and evaluation of the ‘SIKRIBO’ app. This app is used for screening TB-affected persons, data collection, supporting decision-making for health cadres, delivering TB diagnostic test results, treatment evaluation, and education. Evaluation of the app development using the SUS questionnaire.	Health cadres (*n* = 20). Child and adult TB-affected.	The app automatically notifies health cadres whether children and adults meet the criteria for TB after they complete the individual characteristics and TB symptoms.	The development of the ‘SIKRIBO’ app includes six steps. For the evaluation of the usability of the app using the SUS questionnaire, consisting of ten questions, with an average score of 76 (SD, 8.00), to detect active TB in communities with urban and suburban settings	The mHealth app ‘SIKRIBO’, based on Android and a website, was accepted and found to be feasible by health cadres to facilitate screening and early detection of TB cases in the community.	The mHealth app ‘SIKRIBO’ was accepted by health cadres for screening and detection of TB cases in the community. Health cadres stated that the app is user-friendly, not complicated, liked, and instills confidence.	The mHealth app ‘SIKRIBO’ was feasible, easy to use, and easy to learn for data recording, integration, monitoring health cadres in screening implementation, and following up on TB-affected persons for diagnostic examination.
Amanda J. Gupta et al. [48] [2023]	CHWs.	Conducted a case study using an implementation science approach to develop and evaluate the results of a multimodal prospective study of mHealth strategies for implementing TB contact investigations. The assessment included quantitative and qualitative studies to understand feasibility, acceptability, appropriateness, fidelity, and costs.	Household contacts (*n* = 919).	July 2016 to July 2017.	Evaluation of three elements of mHealth implementation strategy in TB contact investigations. First, paper-based records to track and monitor contacts using biometrics. Second, TB screening, testing, and referral algorithm using CommCare. Third, notification of sputum test results using automated SMS.	The mHealth strategy addressed the problem of TB contact investigations by focusing on user-centered development, technology quality, the role of IT experts, devices, and internet access, simplicity of intervention, and continuous monitoring and evaluation.	Biometrics and CommCare Messaging were acceptable strategies for TB contact investigations in the early stages of the trial. However, over time, CHWs’ low perceptions of the appropriateness dimension may have reduced their social image and led to low acceptance of the technology.	Biometrics and CommCare Messaging were feasible strategies in TB contact investigations at the beginning of the pilot phase. However, over time, problems with the system, design, and internet access, lack of IT expertise, complexity of activities, lack of monitoring and evaluation during the process, ultimately reduced the feasibility of mHealth implementation.

TB: tuberculosis; *p*: probability; CI: confidence interval; *ρ*: rho; CHWs: community health workers; LHWs: lay health workers; CHVs: community health volunteers; HCWs: healthcare workers; SMS: short message service; ACF: active case finding; app: application; ODK: open data kit; HTC: high tech computer; CSUQ: computer system usability questionnaire; vs: versus; PPTBMAPP: presumptive pediatric TB mobile android application; CAPI: computer-aided personal interviewing; PAPI: paper and pencil interviewing; NTP: national TB program; SUS: system usability scale; SD: standard deviation; IT: infomation technology.

### Actors

CHWs were the primary implementers across studies, although terminology varied (e.g. community health volunteers (CHVs), lay health workers (LHWs), caretakers, and health cadres), as actors who conduct and/or participate in TB contact investigations (Table 2). Information about the characteristics of the actors is mostly not presented in the papers, from the few papers that do report, it is known that the actors are aged between 17 and 56 years [33,47], secondary education (80%) [33], graduates (70%) [47], training in mHealth interventions [33,43,44,47], trained CHWs [34,43,46,47], incentives [43], and reimbursed [34].

### Actions

The Action in this review is the implementation of mHealth applications and functions in TB contact investigations by CHWs, including (1) targeted communication to persons (*n* = 3), such as ‘CommCare Messaging’ mobile app interventions [34], SMS-based delivery of sputum test results influencing the experiences and behaviors of TB household contacts [49], and mobile phone and SMS interventions [43]; (2) identification and registration of persons (*n* = 2), such as multispectral fingerprint scanner connected to offline software ‘biometrics’ [33], and the use of offline-capable CAPI replaced PAPI [44]; (3) person-centered health records (*n* = 1), such as mobile phone/tablet app interventions designed and developed through Open Data Kit (ODK) and HTC Flyer^TM^ [45]; and (4) healthcare provider decision support (*n* = 2), such as the Presumptive Pediatric TB Mobile Android Application (PPTBMAPP) [46], and the development, intervention, and evaluation of the Android app and website ‘SIKRIBO’ [47]. A detailed description of these actions is presented in Table 2.

**Figure 2.**
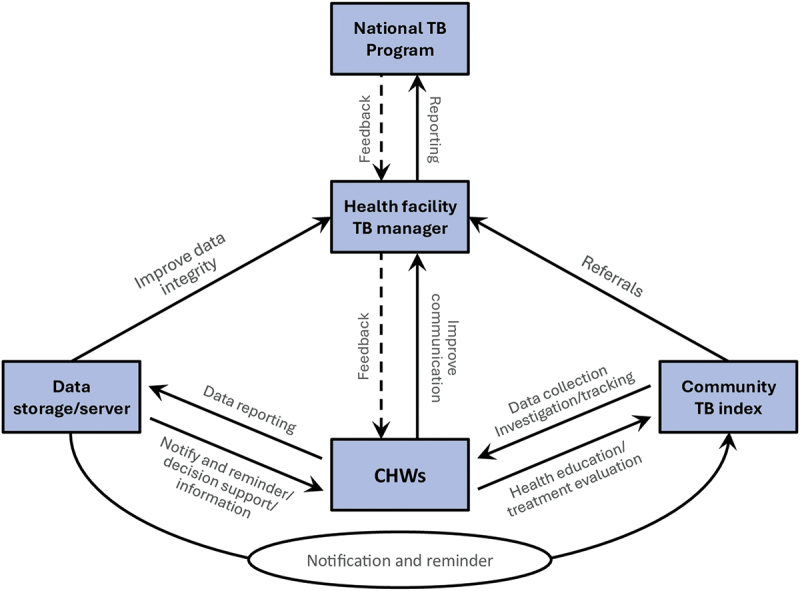
Implementation and function of mHealth in TB contact investigations by CHWs.

On the basis of action dimension, mHealth interventions and functions are integrated with existing health care systems (see Figure 2) and aligned with the WHO operational handbook on the management of tuberculosis in children and adolescents [51]. mHealth technology is utilized for communication with HCWs [46,47], receive automated notifications and reminders about index case home visit schedules from HCWs [46], identification and registration data [33,44], receive decisions from the app regarding the results of identifying TB clinical symptoms and diagnostic tests used as information for result delivery [34,46,47], facilitate referrals and follow-ups to healthcare facilities [34,43,46,49], support treatment evaluation [34,43,47,49], and provide health education to household and close contacts [47,49]. The use of mHealth can accelerate the completion time of investigations [45,46], reduce the waiting time for the delivery of diagnostic test results [43], and shorten reporting time for investigations [45]. Server support and online data storage can enhance data integrity and accessibility for healthcare facilities, TB program managers, and the NTP, thereby supporting monitoring, evaluation, and feedback processes on TB control programs [44].

### Action target

In this review, we grouped these into four action targets (see Table 2), including (1) household contacts TB investigation in adult and pediatric groups [33,34,45,49]; (2) close contact TB investigations, including presumptive TB screening in poor urban settlements for individuals aged ≥ 15 years [43], and individuals aged ≤ 15 years with a history of outpatient visits near TB clinics in hospitals [46]; (3) presumptive TB screening for individuals with remote or low internet access [44]; and (4) screening for TB-affected persons in urban and suburban settings by health cadres [47].

### Temporality

Seven studies were conducted between 2012 and 2021. The mHealth interventions across different technology types were implemented over study periods ranging from 2 to 12 months [33,34,43–46,48,49], whereas one study did not report information on implementation time and study duration [47] (see Table 2).

### Dose

The dose dimension in this review refers to the frequency, duration, intensity, or completeness of implementation of various mHealth interventions and how these aspects are measured. Key indicators include response time (days/week/minute) [34,43,45,49], intervention completion rates (percentage or proportion) [33,34,43,46,47], and the number of identified participants [44] (see Table 2).

### Justification

mHealth interventions in the context of TB contact investigations conducted by CHWs were supported by implementation evidence demonstrating their acceptability and feasibility. These interventions were designed to optimize communication [34,43,48,49], identification and registration [33,44], patient data management [45], and decision-making support [46,47], with detailed information presented in Table 2.

### Barriers and facilitators of mHealth interventions

This review identifies a predominance of barriers over facilitators in the mHealth-supported TB contact investigations, addressing the second research question.

### Barriers

We identified 24 barriers to mHealth implementation experienced by CHWs (Table 3), which were consistent across studies and classified into four categories: CHW-related factors, digital technology systems, service implementation, and TB-affected person factors.

**Table 3.** Barriers and facilitators in mHealth implementation strategy for TB contact investigations.

Categories of barriers and facilitators	Study ID*					
	Uganda	Cambodia	Pakistan	Botswana	Kenya	Indonesia
**BARRIERS**						
**CHW-related factors**						
No employment contract [44]			∏			
Lack of human resources [45]				∏		
Fear of contracting TB [34]	∏					
Inadequate training and/or guidelines on TB contact investigations using mHealth [44,46]			∏		∏	
Lack of soft skills related to TB contact investigations (e.g. ignoring symptomatic individuals, mixed opinion about intervention, difficulty explaining intervention components, limited ability) [33,34,43,47]	∏	∏				∏
Complex deliveries and activities (workload) [34,44]	∏		∏			
Misalignment of work time and implementation schedules (conflicts) [33,49]	∏					
Lack of confidence in intervention (e.g. mistrust of technology, low confirmation of receipt of SMS messages, lack of experience using the app) [33,34,47]	∏					∏
Inconsistency of incentive/reimbursement/compensation [45,48]	∏			∏		
**Digital technology systems**						
Instability of mobile network and low internet connectivity [45,46]				∏	∏	
Malfunction of hardware and software in mHealth components [33,48]	∏					
Lack of end-user needs assessment in development and intervention [33,34]	∏					
Prohibitive development and maintenance costs [48]	∏					
**Service implementation**						
Underestimation of presumptive TB cases likely occurred [43,46]		∏			∏	
Lack of infrastructure data (e.g. location household/map) [34,45]	∏			∏		
Unclear protocol intervention, and record linking technology (paper-based to digital- based) [44]			∏			
Lack of data quality assurance (e.g. inconsistency of data entry, limited data mentoring) [33,44–46]	∏		∏	∏	∏	
Limited field supervision support, and formative evaluation [44,48]	∏		∏			
High implementation costs [43,48]	∏	∏				
**TB-affected person factors**						
Lack of knowledge about TB [34,47]	∏					∏
Inconsistency in interview timing (e.g. busy with work) [43]		∏				
TB-related stigma (anxiety, avoidant) [34,47–49]	∏					∏
Uncertainty of the observed data quality (e.g. symptom screening was conducted through proxies, the Hawthorne effect) [43,45]		∏		∏		
Low literacy and accessibility to digital technology. (e.g. lack of confidence in SMS results in limited follow-up intentions) [34,49]	∏					
**FACILITATORS**						
Involvement of local government and NGOs in the implementation [34,43,44]	∏	∏	∏			
Active engagement in care was discussed with patients [43]		∏				
Operational factors were recognized and addressed during the pilot [45]				∏		
Use of open-source app [34,45,47]	∏			∏		∏
Local input shaped the mHealth intervention design [34,48]	∏					
Patient belief in TB cure and seeking further evaluation [49]	∏					
TB contact investigations involve trained CHWs [33,34,43,46,47]	∏	∏			∏	∏
CHWs training on mHealth technology and problem-solving [33,44,47]	∏		∏			∏
Active community involvement in facilitating implementation strategies [33,43,47,48]	∏	∏				∏
Iterative design incorporates feedback from users [46]					∏	
Regular feedback to measure appreciation and timely intervention [44,46]			∏		∏	

*Country where study was conducted; ∏: yes; CHWs: community health workers; TB: tuberculosis; SMS: short message service; app: application; NGOs: nongovernmental organizations.

#### CHW-related factors

CHW-related factors contributed the largest proportion of implementation barriers (37.5%, *n* = 9), particularly in Uganda (*n* = 6). The most frequently reported barriers were limited soft skills in TB contact investigations [33,34,43,47] and lack of confidence in the intervention [33,34,47]. Additional barriers included unclear criteria for CHW selection [44,45,48], poor communication [33,49], absence of intervention protocols [34,44,46], and inadequate risk mitigation during implementation [34].

#### Digital technology systems

We classify four barriers related to digital technology systems, including unstable cellular network and internet connectivity [45,46], which hindered the uploading of household contact and suspected TB data. Hardware and software malfunctions in mHealth components further reduced effectiveness and discouraged CHWs’ use [33,48]. In addition, the lack of end-user needs assessment during the design, development, and intervention phases limited usability [33,34], while high development and maintenance costs constrained the scalability of digital health systems [48].

#### Service implementation

Three main issues emerged from the six barriers to service implementation. These include data quality barriers, such as the underestimation of presumptive TB cases [43,46], lack of data quality assurance [33,44–46], and lack of infrastructure data [34,45]. Unclear implementation processes, such as a lack of intervention protocols and record-linking technology [44], and limited field supervision and formative evaluation [44,48]. Limited funding, such as high implementation costs [43,48].

#### TB-affected person factors

We identified five barriers associated with TB-affected persons. TB-related stigma was the most frequently reported barrier [34,47–49], and data quality uncertainty was caused by proxy symptom screening and the Hawthorne effect [43,45]. Additional barriers include limited TB knowledge [34,47], inconsistent interview times [43], low literacy, and limited access to digital technology [34,49]. Collectively, these factors increased the burden on CHWs and reduced the functionality of mHealth.

### Facilitators

We identified eleven facilitators, six studies reported more than two facilitators, six studies reported more than two facilitators [33,34,43,44,46,47], while one study identified only one facilitator [49] (see Table 3). For the identified enabling factors, there were two factors highlighted by more than three studies and more than two countries, namely, TB contact investigations involving trained CHWs [33,34,43,46,47], and active community involvement in facilitating implementation strategies [33,43,47,48]. We also identified the relationship between facilitators and barriers, as a study in Pakistan revealed that training on mHealth technology and problem-solving was a facilitator, whereas inadequate training on TB contact investigations using mHealth was a barrier to successful implementation [44].

### Implementation outcomes

Acceptability and feasibility are implementation outcomes that represent CHWs’ perceptions of mHealth interventions in TB contact investigations, based on their functionality, and address the third research question.

### Acceptability

Acceptability refers to CHWs’ perceptions of the appropriateness, appeal, and acceptance of implementation strategies in TB contact investigations [52,53]. Seven studies reported on the acceptability of mHealth implementation (Table 2).

Mobile apps with SMS functionality were generally perceived as acceptable for delivering sputum test results and follow-up instructions. However, over time, there was a decline in the perceived acceptability of one-way SMS due to the low proportion of contacts who received and confirmed SMS messages, incomplete delivery of interventions, and lack of trust in the app [34,48]. Household contacts reported mixed responses, including relief and anxiety, suggesting that SMS should complement rather than replace direct CHW interactions [49].

CAPI interventions were also reported as acceptable for recording and improving data quality in ACF activities conducted in remote or low-internet settings, being perceived as user-friendly, useful, convenient, safe, and effective in managing their use [44]. Similarly, biometric systems were initially well received, enhancing CHWs’ motivation and perceived professional capacity; however, technical malfunctions reduced usability and acceptance over time [33,48].

Mobile and tablet-based apps developed using ODK and HTC Flyer^TM^ were acceptable alternatives to paper-based contact tracing. The apps were perceived as useful, acceptable, and appropriate alternatives to paper-based contact tracing [45]. Likewise, the SIKRIBO app was perceived as user-friendly, uncomplicated, liked, and instilled confidence in TB screening and detection by health cadres [47].

### Feasibility

Feasibility refers to CHWs’ perceptions of whether mHealth interventions in TB contact investigations are practical, implementable, and easy to use [52,53]. Eight studies reported on the feasibility of mHealth implementation (Table 2).

Mobile phone and SMS-based interventions were generally perceived as feasible for supporting ACF by enabling timely notification of diagnostic results, reducing delays, and facilitating treatment initiation and completion [43]. Meanwhile, a mobile app with SMS facilities can be easily used to notify sputum test results and follow-up instructions to household contacts, but did not significantly improve the completeness and yield of home-based TB contact investigations, due to the complexity of the intervention, the lack of guaranteed mobile phone ownership among TB household contacts, and fewer than 20% of SMS messages achieving their intended effect [34,48].

CAPI interventions were feasible in remote areas or with low internet access, improving data completeness and usability in TB screening activities [44]. Similarly, biometric systems were feasible during pilot implementation, with a 70.1% success rate in fingerprint registration, although feasibility declined over time due to technological instability [33].

Mobile and tablet-based apps developed using ODK and HTC Flyer^TM^ were also feasible, easy to use, provided quality information and interfaces, simplified data entry and reception, sped up report writing, and effectively shortened the median contact tracing time compared to paper-based approaches [45]. In addition, the PPTBMAPP intervention improved presumptive TB screening. The app was easy to use, facilitated communication and linkage to TB clinics, and supported daily workloads [46]. Meanwhile, the SIKRIBO app intervention was feasible, easy to use, easy to learn for data recording, integrated, and supported monitoring of health cadres during TB screening and diagnostic follow-up activities [47].

## Discussion

### Implementation strategy

Studies on the use of mHealth in TB contact investigations by CHWs are still developing worldwide, with evidence largely concentrated in a limited number of HBCs, primarily from sub-Saharan Africa and parts of Asia, and mostly conducted in urban or semi-urban contexts.

Given that most included studies were conducted in HBCs that are also LMICs, the findings should be interpreted within similar implementation contexts. Therefore, the generalizability of the review results is not universal, but rather appropriate for contexts with limited resources, CHW-based service models, and similar health system capacities. Differences in study settings, including urban and rural settings, and areas with low internet connectivity, suggest that implementation outcomes are also highly dependent on contextual factors.

WHO recommends increasing high-quality research on digital interventions for TB treatment [54]. Among the nine studies we identified, mHealth interventions in TB contact investigations involving CHWs mostly use hybrid mHealth technologies to meet needs or overcome challenges in the healthcare system. While this integration enhances functional flexibility, it also introduces additional layers of complexity in implementation, particularly in resource-constrained settings where infrastructure and technical support are limited. We highlight four functional classifications that focus on the appropriateness of technology type to their function, while the identification of needs and health system challenges (HSCs) has not been reported. HSCs are a starting point for program planners to track specific issues that need to be addressed and then enable the selection of appropriate DHIs [32,55].

In implementation strategies, mHealth interventions are determined by the actors or stakeholders who use them [56]. With respect to the community, CHWs as actors play a crucial role in TB contact investigations in their communities, as they can ensure a holistic and sustainable approach to help mitigate resource, geographic, TB prevalence, healthcare capacity, social, economic, and cultural barriers [12,57]. However, our findings indicate that the effectiveness of mHealth-supported interventions is not solely determined by CHW involvement but by the alignment between CHW capacity, training, and the technological and system-level support available.

Furthermore, involving trained CHWs can facilitate interventions. However, for new program interventions, consideration should be given to the selection process, detailed explanation of the intervention, provision of training, delivery, risk mitigation, compensation/wage costs, data collection and utilization activities, and employment contracts to increase confidence, responsibility, comfort, and productivity in the operationalization of activities to be carried out before the intervention is performed [58].

All actions in contact investigations, contact tracing, and/or screening conducted by CHWs are based on WHO recommendations [8,51]. Most studies utilized home visits targeting TB index cases, household contacts, and close contacts; however, these actions primarily targeted individuals aged ≥ 5 years, with no reported data for those aged < 5 years. WHO guidelines recommend that household and close contacts aged < 5 years should be prioritized, while individuals aged ≥ 5 years should undergo systematic TB screening [59]. An evidence-based review stated that 83% of children aged < 5 years with TB infection developed TB disease, while infants without tuberculosis preventive treatment (TPT) face an 18% risk of developing TB disease after 2 years of exposure; TPT has been shown to reduce this risk by up to 91% [60].

Regarding temporality and dose, this review shows that almost all studies have gone through an implementation process that supports TB service delivery. From a temporal perspective, mHealth interventions are implemented over a limited period and primarily focused on pilot or trial phases, thus not fully reflecting long-term sustainability or epidemiological impact. From a dose perspective, implementation is based on response time, the level of intervention comprehensiveness, and the number of individuals reached, indicating a diversity of measurement standards used. Dosage can be used as an indicator of adherence and to evaluate the relationship between implementation efforts and outcomes [50]. Temporality and dosage are important dimensions in IR, determining when and how intensively strategies are implemented, enabling accurate measurement and replication. More consistent interventions tend to improve service efficiency, although they do not always correlate directly with clinical outcomes.

In our view, although technical challenges and limitations in the field implementation of mHealth still exist, we argue that mHealth implementation strategies have great potential to address TB contact investigations in HBCs by involving CHWs, particularly in settings with similar health system capacity and digital infrastructure. However, this potential is highly contingent upon contextual readiness, including infrastructure, governance, and human resource capacity, suggesting that mHealth should be viewed as a system-dependent intervention rather than a standalone solution.

Overall, mHealth interventions should clearly define the seven dimensions outlined above. Health program managers and mHealth developers should note that the description of the implementation strategy should be packaged in a detailed protocol or operational guidelines that explain how the intervention will be delivered, while ensuring ongoing evaluation and adaptation to local contextual needs to improve the acceptability and feasibility of mHealth in TB control. Furthermore, despite the sophistication of mHealth, the scalability of interventions when scaled up is influenced by factors beyond the digital technology itself, including health system readiness, digital infrastructure, and ongoing policy support, reinforcing the need for integration into NTP.

### Barriers and facilitators of mHealth interventions

*Barriers*. In CHW-led mHealth interventions, program leaders must carefully select qualified CHWs [61]. However, our review found that none of the included studies reported explicit criteria for CHW selection. Furthermore, lack of communication between HCWs and CHWs impacts CHWs’ perceptions of intervention complexity, reduced trust, and fear of TB exposure, thus limiting mHealth acceptance and use [19,25]. mHealth and CHWs are two different aspects, namely technology and human behavior, so it is necessary to align the design of digital tools with the specific needs of CHWs. Lack of attention to user needs and experiences contributes to the failure of technology adoption [62].

In addition, factors related to the lack of technical support for digital technology systems hinder the acceptability and feasibility of mHealth implementation. Technical support is an important requirement that decision-makers need to consider when implementing district health information systems [63]. Digital health interventions in various other health programs in LMICs show that although mHealth interventions are often well-received, their effectiveness is often limited by systemic and contextual barriers. Reliable mobile network infrastructure and stable internet connectivity are also determinants of the sustainability of mHealth implementation in ensuring the reliability of mHealth technology in its implementation [64], as technology reliability is one of the main extrinsic limiting factors that affect the scale of digital health intervention projects, in addition to network bandwidth and the availability of electricity to charge devices [65], and limited access to smartphones or internet connectivity [66].

Equally important barriers related to privacy and data protection in digital information systems must also be prioritized in their implementation. However, none of the studies we reviewed detailed the data security systems of mHealth interventions. The use of mHealth for data collection, storage, and transmission must be accompanied by data security mechanisms and protect user privacy [67]. Weak regulations and the implementation of data protection in information systems increase the risk of unauthorized access and potential exploitation of patient information. Therefore, integration with national platforms, including improved governance and enforcement of data security and protection regulations [63–66], is crucial to ensure safe and ethical implementation.

TB patient data management remains highly sensitive information within communities in HBCs [68,69]. Informed consent is crucial for data collection, communication, and follow-up using mHealth due to the potential for information leakage, stigma, and other negative social impacts [21,34]. However, the digital literacy gap in TB-affected communities limits patients’ understanding of how their data is used [69]. Therefore, the ethical implementation of mHealth requires robust data security systems, such as encryption and access activation, as well as privacy protection and good governance.

Notably, the high cost of developing, implementing, and maintaining digital health technology impacts organizations’ ability to develop or expand digital technology systems. To obtain significant funding, such as budgets or recovery costs from national financing sources, mHealth implementation must be well-designed and managed throughout the project [67]. Therefore, it is crucial to mobilize additional resources, such as cooperation with national and/or international development partners, considering the limited financial resources in HBCs, to ensure sustainable implementation.

Additional systemic barriers include weak health systems, lack of infrastructure data, and underutilization of information systems, which contribute to incomplete TB data collection and reporting [70] and underestimation of the number of TB-affected persons [71]. Furthermore, the absence of clear intervention protocols, inadequate data support, and limited oversight contribute to suboptimal implementation. To address these issues, mHealth interventions should include clear pre-implementation protocols [29], data quality support for contact tracing [72], and field monitoring and formative evaluation that can provide constructive feedback during the implementation process and make necessary adjustments [31].

The presence of several barriers associated with TB-affected persons in this study represents a complex issue that limits engagement with mHealth initiatives [25,73] and even limits TB patients’ intention to follow-up [68,69]. We highlight factors such as lack of knowledge, stigma, and uncertainty about data quality in symptom screening as requiring special attention in mHealth implementation, as mHealth functions are designed to address these three issues. Therefore, to fulfill its function, the implementation of mHealth must include an educational component, reduce stigmatization, standard protocols for data collection and quality, symptom assessment, and ensure that CHWs are adequately trained to use mHealth [74–78].

*Facilitators*. Several facilitator factors in this study contributed to the successful implementation of mHealth interventions in TB contact investigation by CHWs.

We highlight collaboration between local governments and NGOs as a facilitator in the implementation of mHealth initiatives, particularly the implementation of the DHI policy for TB contact investigation within a health framework that is appropriate and aligned with broader health policies [65], contributes to more effective resource allocation [48], and serves as a significant source of funding [67]. Furthermore, NGOs have a unique ability to engage directly with communities, and their grassroots connections can increase awareness and acceptance of mHealth interventions [48]. Therefore, in the context of mHealth implementation, strategic collaboration is crucial as it facilitates the creation of an environment that encourages mHealth acceptance and obtains resource support, including policies, infrastructure, and financing for the feasibility of mHealth implementation.

Specifically, the involvement of trained CHWs in several studies provided greater value due to resource limitations and ensured that activities could run effectively, efficiently, and accurately [61]. In practice, trained CHWs offer benefits such as better case detection [21,48], improved connectivity with health services [21,79], increased patient engagement and compliance [48], cost efficiency [79], and better data management [72]. In short, the implementation phase is more directed at developing skills related to the effective use and utilization of mHealth technology [80].

Furthermore, active involvement and positive community responses also play a role in facilitating the success of CHW-led mHealth interventions, as they involve CHWs who are part of their communities [48]. This involvement provides benefits in terms of facilitating CHW follow-up on investigation results, such as expediting the referral process, proactive outreach for case detection, and facilitating monitoring and evaluation of TB contact investigations, due to community trust in CHWs.

A critical aspect that needs to be considered and is key to the success of mHealth implementation lies in the design and development of mHealth interventions. We explored the results of several other studies in similar contexts with different programs, which similarly demonstrated that mHealth implementation can be optimized through an iterative design approach that integrates user feedback and local input [65,81,82], uses open-source apps, and provides intensive training for CHWs on mHealth technology and problem-solving [25,82]. Furthermore, regular feedback to gauge user appreciation [81], and identify and address operational issues that arise during the pilot phase can also significantly improve the optimization of mHealth interventions [83].

Meanwhile, mHealth interventions, by involving patients as key stakeholders in shared decision-making, result in personalized, needs-based care plans and instill confidence in TB recovery. In this regard, digital health technology serves as an instrument to complement direct interactions from healthcare providers [49], while still prioritizing patient-centered care in providing quality services [84], and strengthening patients’ psychological recovery by complying with TB treatment follow-up instructions [85]. In addition, information-based interventions at the community level can help reduce stigma and promote support networks for those undergoing treatment [86]. Factors that facilitate the acceptability and feasibility of mHealth interventions in TB contact investigations by CHWs need to be prioritized, while direct barriers are controlled.

### Implementation outcomes

Based on an evaluation framework [87] using an implementation outcomes approach, acceptability and feasibility are indicators for measuring the success of an implementation process or strategy [52,88]. Across studies, acceptability and feasibility were primarily assessed through qualitative perceptions and quantitative implementation indicators, with limited use of standardized IR approaches.

#### Acceptability

Acceptability is a multifaceted construct that reflects the extent to which individuals who provide or receive health care interventions are deemed appropriate on the basis of the cognitive and emotional responses experienced from the interventions performed [89]. The perception of CHWs as individuals who provide and implement mHealth interventions states that mHealth functionality is appropriate, appeal, and acceptable as an implementation strategy in TB contact investigations.

The development of mHealth design and implementation through an iterative process, the integration of local input, and the use of open-source apps play a crucial role in perceived acceptability among CHWs. Furthermore, acceptability can be enhanced through comprehensive technical training on mHealth programs and instruments, regular feedback mechanisms, and proactively addressing operational challenges identified during the pilot phase of the reviewed interventions. However, during this process, several potential barriers can alter CHWs’ perceived acceptability. Therefore, in mHealth interventions, we suggest the need for integration between CHWs and digital health technologies in the context of mHealth implementation acceptability.

The involvement of CHWs in the development and implementation process is an effort to ensure that the mHealth technology designed and used meets their needs and the communities they serve, as well as adding value and supporting their tasks. Technologies that can be accepted and used effectively have the potential to improve the success and sustainability of mHealth implementation [25]. Moreover, health policymakers and IT experts in designing and implementing mHealth must understand why certain behaviors occur and how to develop mHealth implementations that lead to the acceptability of the technology. We argue that the Capability, Opportunity, Motivation, Behavior (COM-B) system framework and Technology Acceptance Model 3 (TAM3) can be used as relevant approaches to address all of these needs. The COM-B system is used to build an understanding of barriers and design implementation strategies for behavior change [90], and TAM3 is used to explain behavioral intentions to use technology, where perceived usefulness and perceived ease of use are crucial in influencing personal attitudes and behaviors towards technology use [91].

#### Feasibility

Based on the results of this review, we argue that mHealth interventions are practical, implementable, and easy to use by CHWs. User retention and active participation in an initiative serve as fundamental indicators in evaluating feasibility and potential positive impact [52]. In addition, the presence of facilitators such as the involvement of trained CHWs, open-source apps, the involvement of local governments and NGOs, active community involvement, active involvement in the treatment discussed with the patient, and the patient’s trust in TB cures also contributes maximally to the feasibility of implementation.

The implementation outcomes are pragmatic when mHealth interventions are acceptable and feasible. Still, this review reveals that there are mHealth interventions that are feasible but not significant in the completion and yield of TB contact investigations. This result highlights a crucial implementation gap, where apparent mHealth feasibility does not always convert into increased efficacy. This is probably because of underlying system limitations, problems with technology dependability, and fluctuations in user involvement. Therefore, optimizing the reliability of mHealth technology, CHW capabilities, and service implementation through assessing needs or issues that need to be addressed, as well as strengthening field supervision support and formative evaluation, which we refer to as moderating factors, can increase the synergy between the action and actor dimensions in implementation feasibility [53].

mHealth reliability should be ensured during operation through system recovery protocols and problem handling in the event of component failure through ongoing quality assurance for mHealth [92] and involving IT experts [93]. Furthermore, the full effect of the intervention during the mHealth implementation process should be evaluated to anticipate the low literacy and accessibility of mHealth in TB-affected persons [94], and it needs to be understood that mHealth is a tool to complement interactions, not to replace direct communication from CHWs.

To enhance acceptability and feasibility, we suggest developing an mHealth implementation strategy for TB contact investigations by CHWs, prioritizing several aspects, including: First, enhancing CHW capacity with a focus on communication and interpersonal skills through a combination of training, mentoring, and ongoing supervision [19,58]. Second, simple design interventions, low-cost, require simple screening instruments, involve few components, and are easy to implement with limited resources [79,90,91]. Third, ensuring the reliability of mHealth technology by involving IT experts and professional designers in mHealth development with a ‘human-centered design’ or ‘design thinking’ approach, which is a method of developing digital technology that prioritizes the desires, needs, and challenges of end users [93,95,96]. Fourth, monitoring and evaluation during implementation using standardized measurement approaches such as acceptability of intervention measure (AIM) and feasibility of intervention measure (FIM) to assess implementation [53], which enables proper detection and handling of issues at every stage of mHealth implementation.

Finally, we argue that implementation outcomes are dynamically and complexly interrelated and tend to change during the CHWs’ process of implementing mHealth. Acceptability of mHealth implementation does not mean that we ignore the feasibility aspect, and vice versa. Therefore, future research and practice should adopt structured implementation planning approaches, such as ‘intervention mapping’, to ensure that mHealth interventions are effective, evidence-based, and responsive to the needs and context of the target population [97]. With the principles of development of transparency, accessibility, scalability, replicability, interoperability, privacy, security, and confidentiality, digital health should be integrated as a core component of system priorities, ensuring that implementation is ethical, safe, secure, reliable, equitable, and sustainable [98].

#### Strengths and limitations

To the best of our knowledge, this is the first review that explores studies with an IR approach to focus on future studies on mHealth implementation strategies in TB contact investigations by CHWs. We conducted a comprehensive literature search, including peer-reviewed literature and gray literature, without restrictions on the year of article publication and study design.

Although these results provide significant contributions, the review we conducted has limitations. First, the number of included studies was relatively small, and the included studies showed substantial heterogeneity in intervention types, implementation settings, study designs, and implementation outcomes, which may limit the generalizability of the findings. Second, a critical appraisal of the articles was not conducted prior to inclusion, as formal quality appraisal is considered an optional step in scoping review methodology, and the included studies may vary in methodological rigor. Third, this scoping review focuses on several papers reporting implementation outcomes and mHealth intervention studies in TB contact investigations involving CHWs in HBCs, so the results of these studies may not be generalizable to low-burden countries of TB.

## Conclusion

CHW engagement in DHIs for TB contact investigation in HBCs is still developing. We identified and mapped seven dimensions of implementation strategy prerequisites, four categories of barriers, five key facilitators, and evidence related to the acceptability and feasibility of a CHW-led mHealth implementation strategy.

This study integrates evidence from diverse biomedical, digital health, and health policy perspectives, serving as a global evidence base and practical insights for designing more context-sensitive mHealth implementation strategies by CHWs. Strengthening CHW capacity, ensuring technology reliability, applying human-centered design, and maintaining continuous monitoring may support more effective implementation. In real-world settings, mHealth interventions may help bridge implementation gaps in CHW-led TB contact investigation by considering health system capacity, infrastructure, and local context. These findings highlight the importance of integrating mHealth into national TB policies and ensuring system readiness for scalability and sustainability in HBCs.

## Supplementary Material

[ZGHA]_OpenScienceForm.docx

## Data Availability

The data that support the findings of this study are openly available in figshare at https://doi.org/10.6084/m9.figshare.30318670.

## Open scholarship

This article has earned the Center for Open Science badges for Open Data and Open Materials through Open Practices Disclosure. The data and materials are openly accessible at https://doi.org/10.17605/OSF.IO/M3A8V
